# Acceleration of wheat breeding: enhancing efficiency and practical application of the speed breeding system

**DOI:** 10.1186/s13007-023-01083-1

**Published:** 2023-11-04

**Authors:** Jin-Kyung Cha, Hyeonjin Park, Changhyun Choi, Youngho Kwon, So-Myeong Lee, Ki-Won Oh, Jong-Min Ko, Soon-Wook Kwon, Jong-Hee Lee

**Affiliations:** 1 Department of Southern Area Crop Science, Rural Development Administration, National Institute of Crop Science, Miryang, 50424 Republic of Korea; 2 Rural Development Administration, National Institute of Crop Science, Wanju, 55365 Republic of Korea; 3 Rural Development Administration, Jeonju, 54875 Republic of Korea; 4 Department of Plant Bioscience, Pusan National University, Miryang, 60463 Republic of Korea

**Keywords:** Breeding, Generation acceleration, Speed breeding, Speed vernalization, Wheat

## Abstract

**Background:**

Crop breeding should be accelerated to address global warming and climate change. Wheat (*Triticum aestivum* L.) is a major food crop. Speed breeding (SB) and speed vernalization (SV) techniques for spring and winter wheat have recently been established. However, there are few practical examples of these strategies being used economically and efficiently in breeding programs. We aimed to establish and evaluate the performance of a breeder-friendly and energy-saving generation acceleration system by modifying the SV + SB system.

**Results:**

In this study, a four-generation advancement system for wheat (regardless of its growth habits) was established and evaluated using an energy-efficient extended photoperiod treatment. A glasshouse with a 22-hour photoperiod that used 10 h of natural sunlight and 12 h of LED lights, and minimized temperature control during the winter season, was successful in accelerating generation. Even with one or two field tests, modified speed breeding (mSB) combined with a speed vernalization system (SV + mSB) reduced breeding time by more than half compared to traditional field-based methods. When compared to the existing SV + SB system, the SV + mSB system reduced energy use by 80% to maintain a 22-hour photoperiod. Significant correlations were found between the SV + mSB and field conditions in the number of days to heading (DTH) and culm length (CL). Genetic resources, recombinant inbred lines, and breeding materials that exhibited shorter DTH and CL values under SV + mSB conditions showed the same pattern in the field.

**Conclusions:**

The results of our SV + mSB model, as well as its practical application in wheat breeding programs, are expected to help breeders worldwide incorporate generation acceleration systems into their conventional breeding programs.

**Supplementary Information:**

The online version contains supplementary material available at 10.1186/s13007-023-01083-1.

## Background

Wheat (*Triticum aestivum* L.) is a major global food crop that accounts for 20% of the calories and proteins in the human diet [1]. Wheat yield, stability, and disease resistance have gradually improved as a result of breeding progress [2]. Breeding initiatives have also successfully addressed global food security concerns, resulting in a doubling of wheat production within 20 years [3]. However, the ongoing effects of global climate change necessitate the development of crop varieties that can adapt to the changing environmental conditions [4]. The generation time of new cultivars is a crucial limiting factor in their rapid development [5]. Traditional breeding methods often take one to two decades to develop a new cultivar, involving processes such as crossing, selection, and field-based testing [6, 7], as well as a substantial amount of field space and manpower.

To address this issue, a speed breeding (SB) system with an extended photoperiod (22 h) was developed, allowing for up to six generations of spring wheat and spring barley per year [8]. An advanced speed vernalization (SV) system was also developed [9]. When the SV system is combined with the SB system (SV + SB), up to five generations of winter wheat and winter barley can be grown per year at relatively higher vernalization temperatures. The SV + SB system is particularly useful for shortening generation times in crosses between spring and winter wheat cultivars while effectively satisfying the vernalization requirements across diverse genetic resources. The SV + SB system has enabled the use of genetic resources with varied genetic backgrounds in generation-acceleration systems for breeding, removing growth-habit restrictions.

The SV + SB system contributed to a reduction in breeding time. However, to optimize breeding efficiency, adequate phenotypic and genotypic selection must be carried out during breeding cycles. Under SB conditions, resistance to multiple diseases, including tan spots, stripe rust, leaf rust, and crown rot, can be evaluated 4–6 times using a large numbers of plant materials, whereas in field conditions, it can only be conducted once a year [10–13]. The SB and SV + SB systems can also be used in conjunction with marker-assisted selection (MAS) and genomic selection (GS) to stack key target genes or traits [13]. Nonetheless, certain agronomic traits need to be evaluated more effectively and economically using direct phenotypic screening methods [14]. For example, flowering time and plant height can be visually evaluated by breeders at the time of harvesting [15]. This effectively decreases the size of breeding material while reducing the time and cost of genotyping breeding lines over generations.

Wanga et al. [16] emphasized that certain key aspects must be addressed for the SB system to be effectively used by plant breeders worldwide. First, experts were proficient in using the SB system for breeding purposes. Second, sophisticated facilities capable of regulating temperature and light conditions must be set up. Finally, the facilities must have a dependable supply of water and electricity to operate. Two growth rooms capable of controlling temperature and light conditions were required for plant growth in the SV + SB system. One is the vernalization room, which is kept at 8–10 °C with a photoperiod of 22 h of light [9]. These vernalization conditions must be maintained to meet the vernalization requirements of varied genetic resources within breeding programs. In contrast, the SB room, which is set at 22 °C during the day (22 h) and 17 °C at night (2 h) [17], can be modified to take advantage of natural environmental conditions to save energy and money on the installation and management of the controlled indoor growth room. Cha et al. [7, 18] reported a significant reduction in the days to heading (DTH) for spring wheat and triticale using a 22-h photoperiod with 10 h of natural sunlight and 12 h of artificial lighting.

By adapting the SV + SB system, we established and tested the efficacy of a breeder-friendly and energy-saving generation acceleration system that enables four generations of spring and winter wheat per year. A large amount of breeding material was used to test the modified SB (mSB) system combined with SV (SV + mSB), which uses a glasshouse and natural sunlight, to ensure that this system can be applied to breeding routines. To ascertain whether intuitive phenotypic selection could be carried out under the SV + mSB settings, phenotypic correlations between the field and SV + mSB conditions were also assessed.

## Results and discussion

### Glasshouse with minimized artificial control enables speed breeding in wheat

**Fig. 1:**
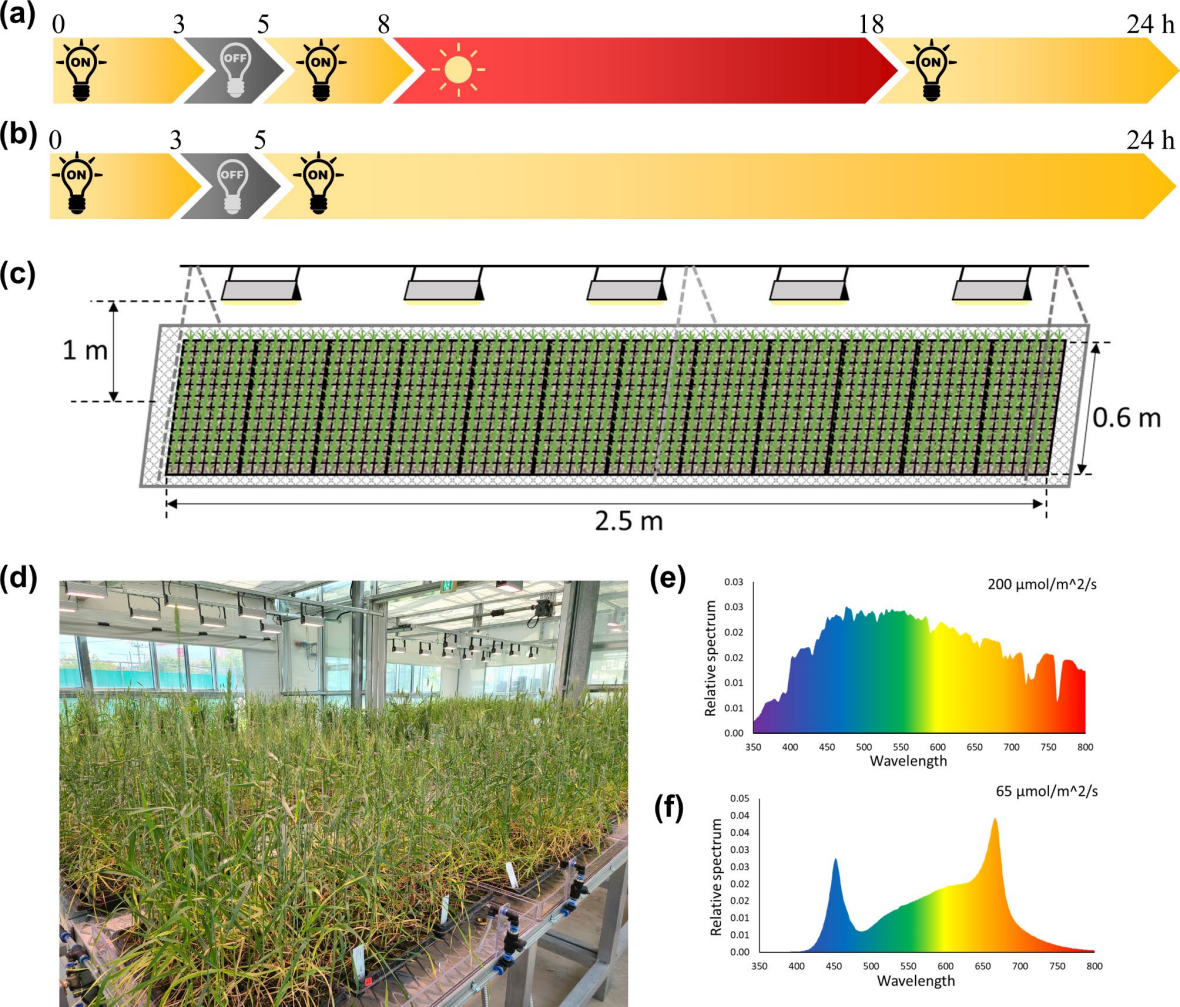
Modified speed breeding (mSB) condition using natural sunlight Comparison of light conditions between (a) mSB in a glasshouse, and (b) original speed breeding in growth room. Schematic (c) and photograph (d) of light installations in a glasshouse. Light intensity and quality in the glasshouse in the day (e) and at night (f)

The photoperiod for mSB conditions was 22 h per day, consisting of 10 h of natural sunlight and 12 h of LED light (Fig. 1a). Compared to prior research that used LED lamps or high-pressure sodium vapor lights throughout a 22-h photoperiod (Fig. 1b) [8, 9], the mSB technique substantially reduced dependency on artificial lighting by harnessing natural sunlight. The mSB facility in the glasshouse, equipped with 60 LED lamps, accommodated 144 trays, whereas the conventional SB growth room accommodated 120 trays with 210 LED lights (Fig. 1c and d, Table S1). As a result, despite its ability to accommodate a larger number of trays, the mSB condition used 80% less energy for LED lighting than the SB condition. During the day, the photosynthetic photon flux density (PPFD) from the shelf to the lights was 200 µmol/m²/s (Fig. 1e), while at night it was 65 µmol/m²/s (Fig. 1f). Although this light intensity was lower than that of SB rooms in previous studies, which ranged from 350 to 500 µmol/m²/s [8, 9, 19, 20], no significant differences in days to heading (DTH) or grain number per spike (GN) were observed between the SV + mSB and SV + SB conditions (Fig. S1a and b). This may be because plant growth is influenced by various conditions, including light intensity, light spectrum, temperature, and CO_2_ concentration [13, 17, 21–23]. Bhatta et al. [13] reported that higher plant height and GN were observed in a glasshouse than in a speed-breeding room, although this was the result of delayed DTH in a glasshouse with a natural photoperiod. Because a short DTH and sufficient GN are the main factors for accelerating wheat generation, the mSB condition is considered suitable for decreasing the generation time while maintaining minimal plant growth and enough grains.

### Four-generation advancement system for wheat breeding program

**Fig. 2:**
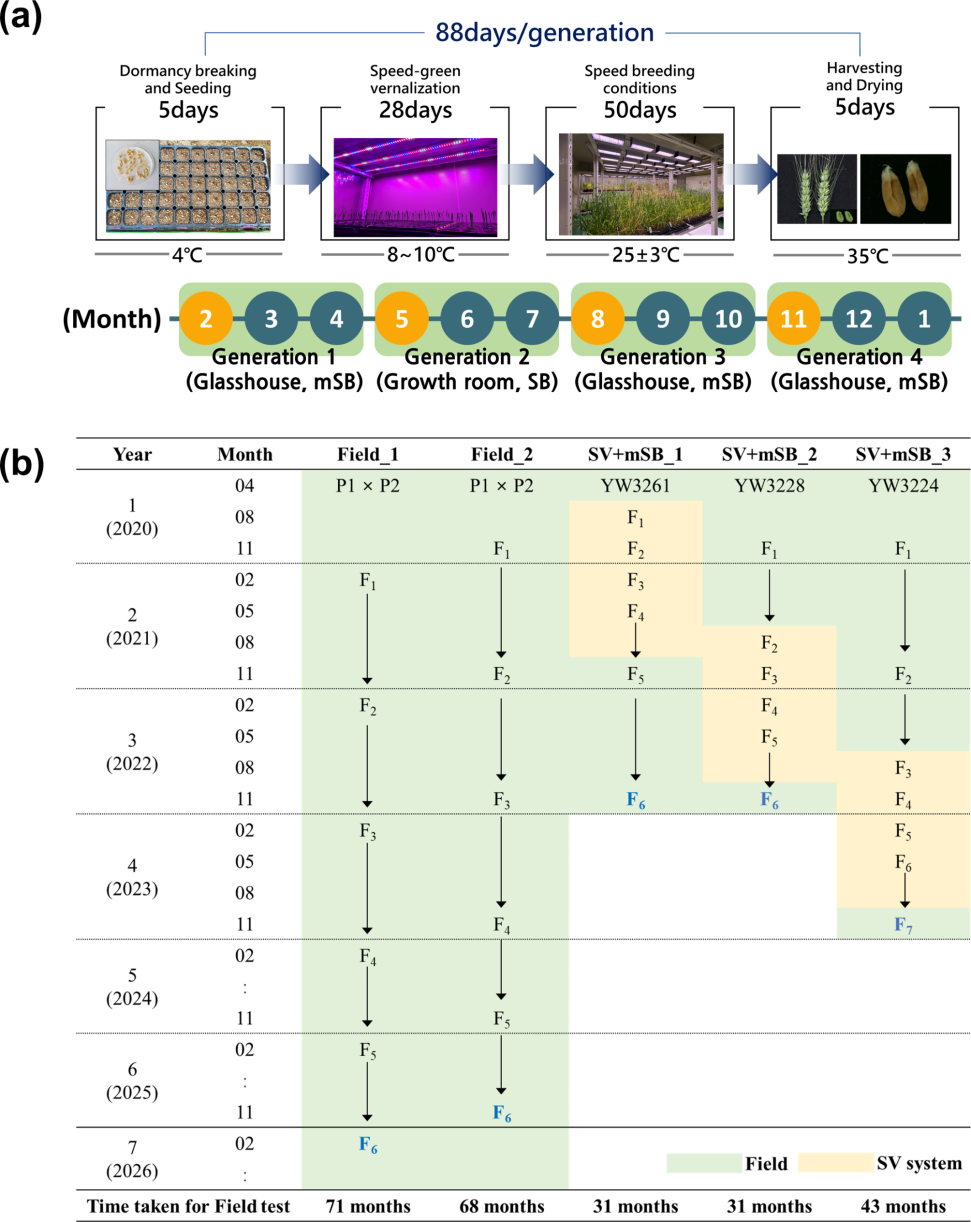
Modified speed breeding condition combined with speed vernalization (SV + mSB) system enables four generations per year for rapid development of new wheat lines (a) Diagram of SV + mSB conditions. The months marked with yellow indicate one week of speed vernalization (SV) treatment, while blue indicates speed breeding (SB) or modified speed breeding (mSB) conditions. (b) Schematic representation for comparing the generation advancement in traditional field and three SV + mSB conditions. The blue bold generation applies to observed yield trials. Field_1 and 2 represent spring and winter wheat, respectively. The numbers followed by SV + mSB indicate each model using the SV + mSB system starting from different generations. YW3261: Jokyoung/Joongmo2008//Baekkang/m Joongmo2008, YW3228: Milyang46/Garnet, and YW3224: Keumgang/Joongmo2008. Jokyoung, Baekkang and Garnet are spring cultivars, and the others are winter cultivars

Figure 2a describes the four-generation advancement system using the SV + mSB conditions. The glasshouse has been used for three generations of advancement, with only one cycle requiring a controlled growth room. The timeline was altered to start in February, instead of January, to reduce the duration of wheat growing in the glasshouse during the summer season. Because the temperature in Korea is highest from June to August (Table S2), it was preferable to execute the second cycle (May–July) in the growth room. The third cycle (August–October) includes the hottest month of the year, August, yet four weeks of SV treatment can limit plant growth in the glasshouse at this time. Although the second cycle can be conducted in a glasshouse, as a result of high temperatures in the heading and flowering stages, GN and plant fertility significantly decreased compared with the other cycles (Fig. S1, Table S3 and S4). The time schedule can be adjusted according to the climate conditions in each country and region, and entire cycles can be conducted under glasshouse conditions if the temperature is relatively low throughout the summer. According to Chat et al. [9], five generations of spring × winter wheat population can be developed in 12 months. However, four generations can be accelerated in some winter wheat cultivars. As numerous genetic resources with varying development behaviors are used in breeding programs, this four-generation advancement system can be optimized for wheat breeders to use on a regular basis.

In observed yield trials (OYT), the SV + mSB reduced the time from artificial crossing to sowing wheat grains in the field by more than half when compared to conventional field breeding systems (Fig. 2b). Under field conditions, because one generation of spring and winter wheat can be developed per year, the F_6_ generation typically takes 68–71 months to develop. When large breeding materials from various cross combinations were developed using the SV + mSB system, four generations could be stably advanced in one year, with additional time for sufficient seed maturity before evaluating them in the field (Fig. 2b, Table S5). The breeding time in the SV + mSB_1 and SV + mSB_2 models, which accelerated generation from F_1_ and F_2_, respectively, was shorter than in the SV + SB _3 model, which originated from F_3_ (see the schematic for each model in Fig. 2b). However, to maintain sufficient segregation of the F_2_ population size and achieve targeted gene-recombinant lines [24, 25], the SV + mSB_3 model would be suitable for field breeding programs. Because wheat grown in pots yields more grains than wheat grown in trays under SB conditions [7], pots would be more efficient for developing the last generation to be sown in the field as lines.

### Phenotypic selection in the SV + mSB system

**Fig. 3:**
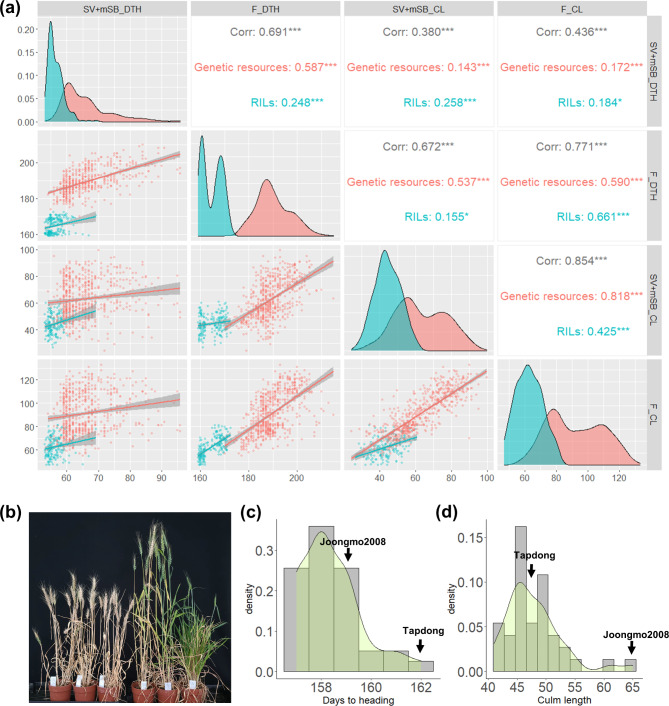
Phenotypic selections can be conducted under the modified speed breeding combined with the speed vernalization condition (SV + mSB). (a) Correlations of days to heading (DTH) and culm length (CL) between the SV + mSB and field conditions. Genetic resources: a total of 609 wheat cultivars collected worldwide; RILs: Jokyoung×Joongmo2008 derived 184 recombinant inbred lines. (b) The phenotypic difference in Joongmo2008*2/Tapdong BC_1_F_4_ lines under the SV + mSB condition. The left three individuals represent selected lines with early heading and short CL, while the right three lines were eliminated due to their delayed DTH and long CL. The distribution of DTH (c) and CL (d) in Joongmo2008*2/Tapdong BC_1_F_5_ 37 lines evaluated in field

Agronomic traits, such as DTH, plant height, and spike length (SPL) are highly heritable under a variety of field conditions [26–28]. Spring wheat grown in the SB condition also showed high heritability for such agronomic traits [29]. We investigated the correlation between SV + mSB and field conditions for several agronomic traits to enable phenotypic selection in SV + mSB conditions for breeding materials obtained from both spring and winter wheat cultivars. When the data from SV + mSB and field conditions were compared with 609 genetic resources and 184 Jokyoung × Joongmo2008 recombinant inbred lines (RILs), high correlations between DTH (*r* = 0.691***) and culm length (CL) (*r* = 0.854***) were found (Fig. 3a). RILs had a weaker correlation in both DTH and CL than the genetic resources because of their lower phenotypic diversity and narrower distribution. SPL also showed a significant correlation with SV + mSB and field conditions, whereas awn length (AL) and spikelet number per spike (SPN) showed no significant correlation between these two conditions (Fig. S2).

Based on these findings, visual phenotypic selection for DTH and CL was applied on the Joongmo2008*/Tapdong (winter-type × winter-type) BC_1_F_4_ lines under the SV + mSB conditions (Fig. 3b, Fig. S3). Lines with early heading and short CLs were harvested and sown for the next generation. Under field conditions, all BC_1_F_5_ lines had earlier DTH than Tapdong (late heading and short culm length), and shorter CL than Joongmo2008 (early heading and long CL) (Fig. 3c and d). This result indicates that efficient phenotypic selection can be conducted under the SV + mSB conditions to reduce the population size and labor required for evaluating breeding lines in the field.

However, generation acceleration and phenotypic selection in the SV + mSB conditions remain limited owing to indoor testing across multiple generations. The annual evaluation of breeding lines in local fields allows better-adapted cultivars to be selected [30–32]. Borlaug’s shuttle breeding method allows for generation acceleration and natural selection through cultivation in diverse environments [33, 34]. Furthermore, the selection of major yield-related agronomic traits, such as tiller number, number of grains per spike, and thousand grain weight, cannot be conducted under the SV + mSB conditions. As a result, although the SV + mSB system can substantially reduce breeding time and input energy on its own, it still requires the use of modern breeding technologies such as MAS and GS. Because numerous markers have been developed for selecting breeding targets, including major yield-related traits, MAS and GS would helpmaximize genetic gain and enable the selection of lines that are adaptable to each cultivation environment [17, 35, 36].

## Conclusion

We modified SB conditions in this study to create a breeder-friendly and energy-saving generation acceleration system. The SV + mSB system uses 10 h of natural sunlight and 12 h of LED light, resulting in an 80% reduction in energy consumption compared to the conventional SV + SB system. Four generations of wheat can be developed using the SV + mSB system as standard procedure in breeding programs, enabling the production of a sizable amount of breeding material. When the SV + mSB system was combined with field tests, the breeding time was reduced by more than half compared to the traditional field-based breeding method. Visual phenotypic selection was effective in the SV + mSB condition. Genetic resources, RILs, and breeding lines that had shorter DTH and CL under SV + mSB conditions also had shorter DTH and CL under field conditions. The findings of this study, which involved the successful use of phenotypic selection while accelerating the development of four generations of bulk breeding materials, are expected to serve as a model for breeders worldwide. However, it is still difficult to evaluate major yield-related traits under SV + mSB conditions. Therefore, developing and utilizing gene-specific markers that match the genotype and phenotype in each field condition would enhance both speed and efficiency for wheat breeding.

## Materials and methods

### Modified speed breeding condition set up in a glasshouse

A glasshouse with shelving for plant trays was used for the testing of mSB conditions. LED lights (Full Spectrum LED PLANT 50WB_SPOT_Full; Yunlighting Co., Namyangju, Korea) were mounted at a height of one meter from the shelves where the plants were placed, with five LED lights per 1.5 m^2^ in the modified speed breeding (mSB) glasshouse (Fig. 1c, d). The light composition and intensity were measured using an RS-3500 field portable spectroradiometer (Spectral Evolution Inc., Haverhill, MA, USA) and an LI-250 light meter (LI-COR Biosciences, Lincoln, NE, USA), respectively. Each shelf held twelve 72-cell trays, and the glasshouse had 12 shelves altogether. The total area of the glasshouse was 115.5 m^2^ (Table S1). The glasshouse is located at the National Institute of Crop Science (NICS), Rural Development Administration (RDA), Miryang, Republic of Korea (35°29′32.9′′N, 128°44′33.4′′).

The temperature inside the glasshouse was adjusted by opening and closing the windows, without a cooling system, and a heating system was used for 192 days per year, when the mean minimum temperature was less than 10 °C (late October to March). A Testo 174 H logger (Testo Industrial Services GmbH, Kirchzarten, Germany) was used to record the temperature inside the glasshouse. The weather data supplied by the Korea Meteorological Administration Open MET Data Portal (https://data.kma.go.kr/) was used to collect outdoor temperature data.

### Seeding and vernalization treatment

Using the SV method developed by Cha et al. [9], the vernalization room was set to 8 °C to meet the vernalization requirements of all wheat cultivars, genetic resources, and breeding materials included in this study. All the seeds were moistend and chilled at 4 °C for 3 to 4 days, then moved to the vernalization room shortly after sowing. The 72-cell trays (W 27 cm, L 58 cm) were used for generation advancement with the single-seed descent method, whereas 250 ml pots were used for artificial crossing and generation advancement of F_1_ plants. The trays and pots were filled with a mixture of commercial paddy rice soil (Punong Co. Ltd., Gyeongju, Korea) and horticultural soil (Seoul-Bio Co. Ltd., Eumseong, Korea) in a 2:1 ratio. All plants were moved to the SB or mSB conditions after four weeks of vernalization. The SB condition was the same as that used by Cha et al. [9], which maintains the 22 °C during the 22 h of day, and 17 °C during the 2 h of night.

### Plant materials and growth evaluation

To compare the growth characteristics under SV + SB and SV + mSB conditions with different seeding dates, ten wheat cultivars, including both spring- and winter-type, were evaluated using five plants per cultivar (Table S4).

To evaluate the correlation between agronomic traits in SV + mSB and field conditions, 609 worldwide wheat genetic resources reported by Min et al. [37] and 184 RILs derived from a cross between Jokyoung (spring-type) and Joongmo2008 (winter-type) were sown in 72-cell trays with six plants per cultivar. The detailed information of the worldwide wheat accession is reported by Kang et al. [38]. Field data on genetic resources were collected over three years (2018–2020), whereas RILs were evaluated over two years (2021–2022). Tottman et al. [39] detailed the recording of the heading stage date (GS59). The culm length, spike length, awn length, and spikelet number per spike were determined using the RDA Standard Evaluation Manual for Agricultural Experiments and Research [40].

To assess the impact of phenotypic selection under SV + mSB conditions, 37 Joongmo2008*2/Tapdong BC_1_F_5_ lines were developed, as shown in Fig. S3. Visual selection under SV + mSB conditions was conducted in the BC_1_F_5_ generation. Phenotypic evaluation in the field was carried out in 2022.

### Statistical anaylsis

RStudio (version 1.4.1717; RStudio, PBC, Boston, MA, USA) was used for all statistical analyses. Correlation analysis, analysis of variance, and Duncan’s multiple tests were conducted using ggplot2, agricolae, and GGally packages.

### Electronic supplementary material

Below is the link to the electronic supplementary material.

Supplementary Material 1

## Data Availability

The datasets used and/or analyzed in the current study are available from the corresponding author upon reasonable request.

## Abbreviations

AL: awn length
CL: culm length
DTH: days to heading
GN: grain number per spike
GS: genomic selection
MAS: marker-assisted selection
mSB: modified speed breeding
OYT: observed yield trials
PPFD: photosynthetic photon flux density
RILs: recombinant inbred lines
SB: speed breeding
SPL: spike length
SPN: spikelet number per spike
SV: speed vernalization
