# Supplementary material for: Acceleration of wheat breeding: enhancing efficiency and practical application of the speed breeding system
Source: Plant Methods. 2023 Nov 4;19:118. doi: 10.1186/s13007-023-01083-1 (PMC10625215; doi:10.1186/s13007-023-01083-1)
Supplement: Supplementary file 1 — Supplementary Material 1 [file 13007_2023_1083_MOESM1_ESM.docx]

**Table S1.** Comparison of the annual energy and cost inputs for operating a growth room for speed breeding conditions and a glasshouse for modified speed breeding conditions.

| Control parameter | Condition | Growth room | Glasshouse |
| --- | --- | --- | --- |
| Capacity | Area | 27.5 m^2^ | 115.5 m^2^ |
|  | No. of layers | 2 | 1 |
|  | No. of trays | 120 | 144 |
| Light | Type | LED lights | Sunlight + LED lights |
|  | No. of LED lamps | 210 | 60 |
|  | Operating time (h/day) | 22 | 12 |
|  | Energy use (kW/day) | 284.6 | 44.4 |
| Temperature control | Parameter | Heating & Cooling | Heating |
|  | Energy type | Electricity | Diesel |
|  | Operating days per year | 365 | 192 |
|  | Energy use per year | 27,265.5 kW | 144,348 L |

**Table S2.** Monthly temperatures (°C) in the field and inside the glasshouse. The correlation is significant at the 0.001 level.

| **Year** | **Month** | **Average** | | **Mean maximum** | | **Mean minimum** | |
| --- | --- | --- | --- | --- | --- | --- | --- |
|  |  | **Glasshouse** | **Field** | **Glasshouse** | **Field** | **Glasshouse** | **Field** |
| 2020 | May | 23.5 | 18.9 | 31.2 | 25.3 | 16.7 | 13.0 |
|  | June | 27.4 | 23.9 | 35.7 | 30.2 | 21.0 | 18.7 |
|  | July | 26.4 | 23.1 | 32.7 | 27.0 | 22.2 | 20.2 |
|  | August | 31.1 | 27.8 | 38.5 | 32.5 | 26.2 | 24.2 |
|  | September | 24.9 | 21.0 | 32.6 | 25.9 | 19.9 | 16.7 |
|  | October | 20.1 | 14.6 | 28.7 | 21.8 | 14.6 | 8.7 |
|  | November | 21.8 | 9.3 | 27.7 | 16.2 | 19.0 | 3.1 |
|  | December | 23.1 | 1.2 | 26.8 | 8.0 | 18.8 | −4.3 |
| 2021 | January | 23.2 | 0.4 | 27.7 | 7.0 | 18.9 | −5.3 |
|  | February | 23.7 | 4.3 | 30.1 | 11.7 | 19.5 | −2.4 |
|  | March | 22.1 | 10.1 | 28.0 | 16.9 | 18.7 | 3.9 |
|  | April | 22.2 | 14.0 | 30.3 | 21.2 | 17.4 | 7.1 |
|  | **Average** | **24.1** | **14.1** | **30.8** | **20.2** | **19.4** | **8.6** |
|  | **Correlation (*r*)** | **0.618** | | **0.714** | | **0.518** | |

**Table S3.** Comparison of plant flowering and seed production in the growth room (GR) and the glasshouse (GH) with different seeding date. GR and GH1: Seeded on 8^th^ April; GH2: 6^th^ May; and GH3: 20^th^, May in 2020.

| Condition | No. of plants | No. of plants with  no flowering | No. of sterile plants |
| --- | --- | --- | --- |
| GR | 50 | 1 | 1 |
| GH1 | 50 | 0 | 0 |
| GH2 | 50 | 6 | 15 |
| GH3 | 50 | 2 | 4 |

**Table S4.** List of wheat cultivars with different seeding dates evaluated under the modified speed breeding condition.

| Cultivar | Growth habit | It no. |
| --- | --- | --- |
| Owolsomaek | Spring | 14380 |
| Giok 27 | Spring | - |
| Jokyoung | Spring | 213249 |
| Goso | Spring | 332393 |
| Keumgang | Winter | 213100 |
| Younbaek | Winter | 227127 |
| Hojoong | Winter | 311644 |
| Sotaek | Winter | 115977 |
| Jaseon 1 | Winter | - |
| Chokwang | Winter | 116143 |

**Table S5.** Utilization of the modified speed breeding condition combined with the speed vernalization conditions in wheat breeding program. The days per generation were calculated as the duration from seeding to harvesting, with an additional week accounted for breaking seed dormancy.

| **Year** | **No.** | **Seeding (mm/dd)** | **Harvesting (mm/dd)** | **Days** **/generation^1)^** | **No. of cross combination** | | | | **No.**  **of lines** |
| --- | --- | --- | --- | --- | --- | --- | --- | --- | --- |
|  |  |  |  |  | **F_1_** | **F_2_** | **F_3–7_** | **Total** |  |
| 2020 | 1^st^ | 02/24 | 05/20 | 93 | 1 | 8 | 4 | 13 | 2,418 |
|  | 2^nd^ | 05/27 | 08/04 | 76 | 5 | 0 | 9 | 14 | 3,581 |
|  | 3^rd^ | 08/19 | 11/20 | 100 | 10 | 1 | 3 | 14 | 770 |
|  | 4^th^ | 11/03 | 01/29 | 94 | 2 | 2 | 8 | 12 | 2,881 |
| 2021 | 1^st^ | 02/05 | 04/26 | 87 | 7 | 5 | 9 | 21 | 6,100 |
|  | 2^nd^ | 05/06 | 07/22 | 84 | 0 | 5 | 16 | 21 | 7,600 |
|  | 3^rd^ | 07/29 | 11/01 | 102 | 0 | 0 | 29 | 29 | 8,420 |
|  | 4^th^ | 11/09 | 02/08 | 98 | 0 | 5 | 15 | 20 | 5,820 |
| 2022 | 1^st^ | 02/17 | 05/06 | 85 | 0 | 0 | 20 | 20 | 5,555 |
|  | 2^nd^ | 05/13 | 07/29 | 84 | 0 | 0 | 27 | 27 | 4,611 |
|  | 3^rd^ | 08/05 | 10/28 | 91 | 13 | 0 | 15 | 28 | 4,290 |
|  | 4^th^ | 11/03 | 01/30 | 95 | 0 | 5 | 15 | 20 | 11,641 |
|  | **Average** | | | **91** | **-** | **-** | **-** | **20** | **5,307** |


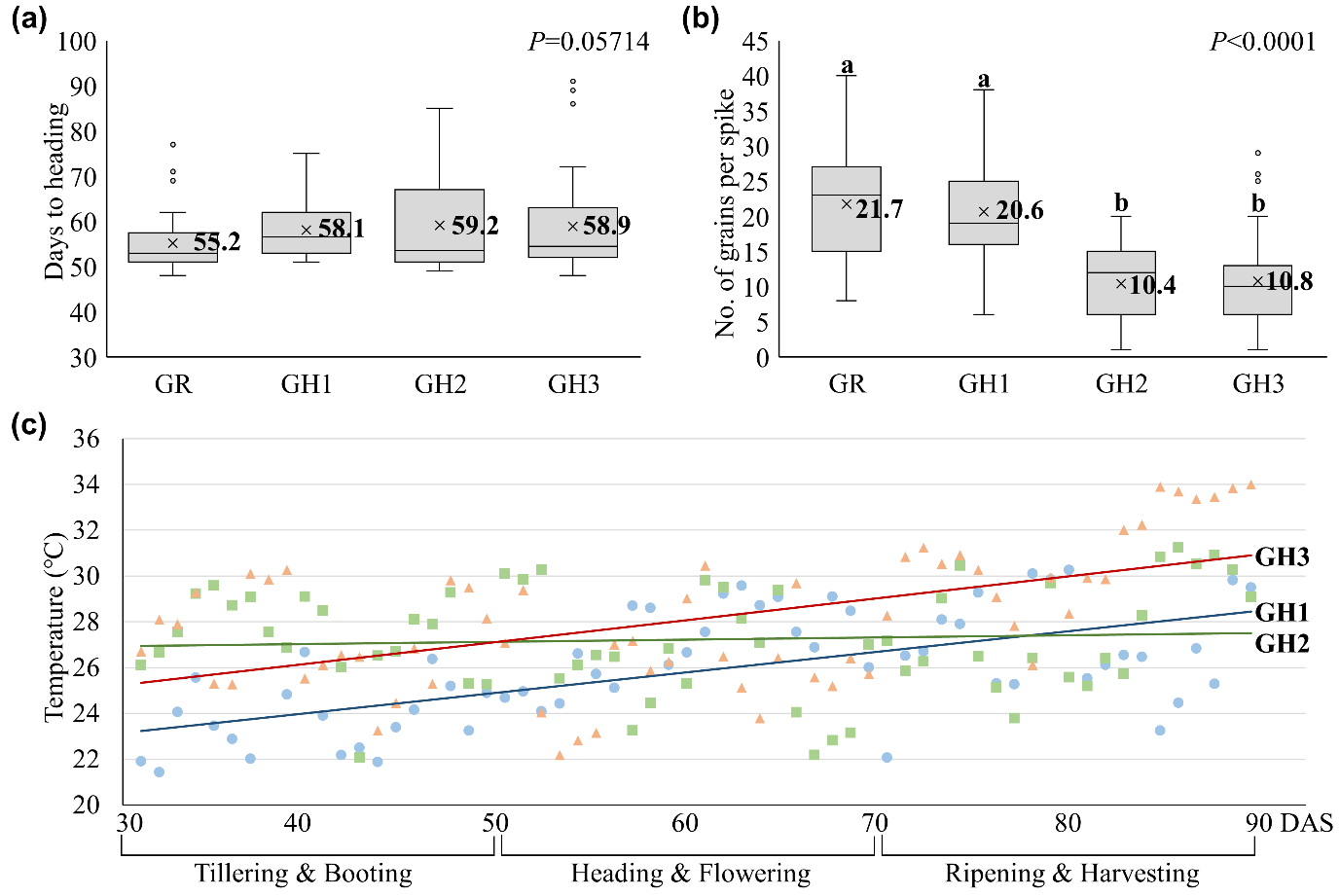


**Figure S1.** Comparison of days to heading (a) and number of grains per spike (b) in growth room (GR) and glasshouse (GH) with different seeding dates. GR and GH1: Seeded on 8^th^ April; GH2: 6^th^ May; and GH3: 20^th^, May in 2020. Different letters indicate significance according to Duncan’s multiple test. (c) Average daily temperature inside the glasshouse during wheat growth stages depending on the sowing time. DAS: days after seeding.


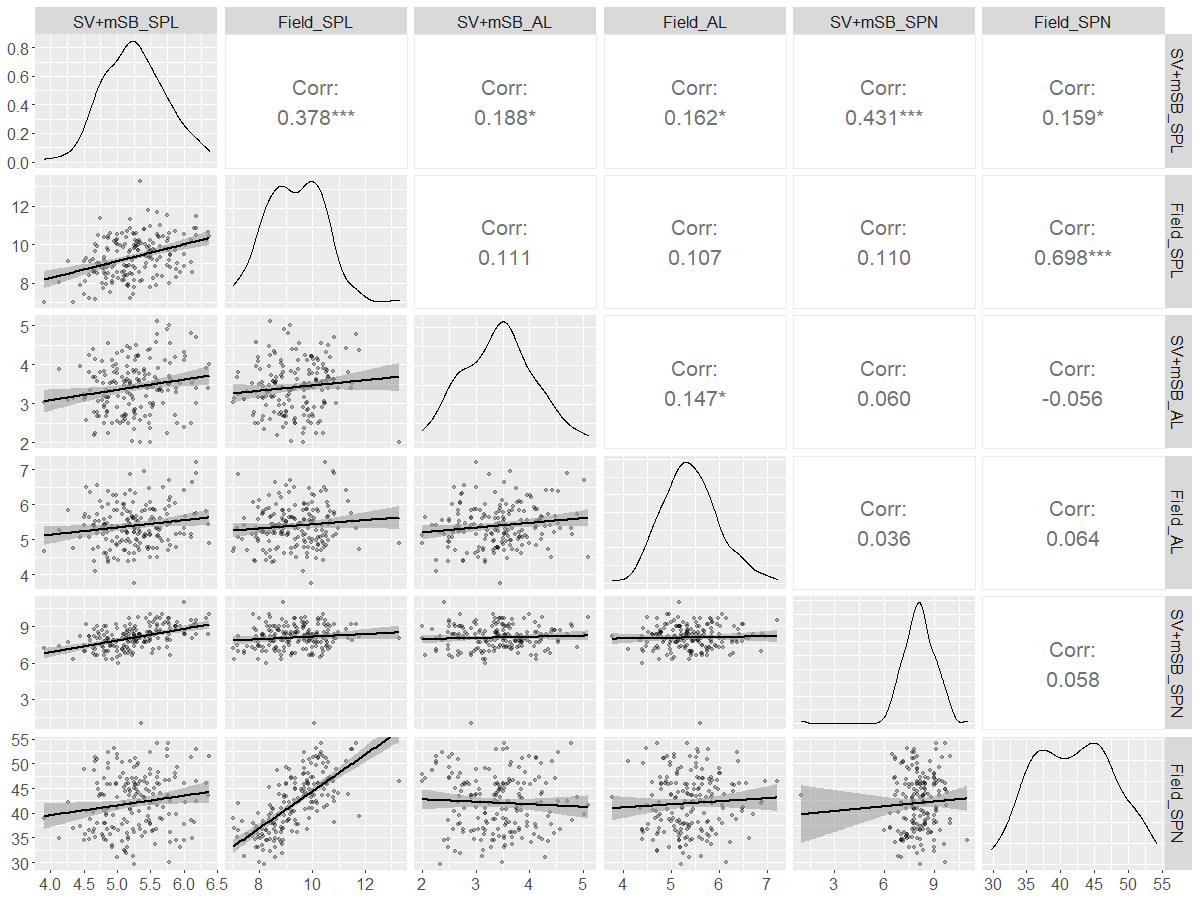


**Figure S2.** Correlations of agronomic traits of Jokyoung × Joongmo2008 184 RILs evaluated under the speed vernalization + modified speed breeding (SV+mSB) and field conditions. SPL: spike length, AL: awn length, SPN: spikelet number per spike.


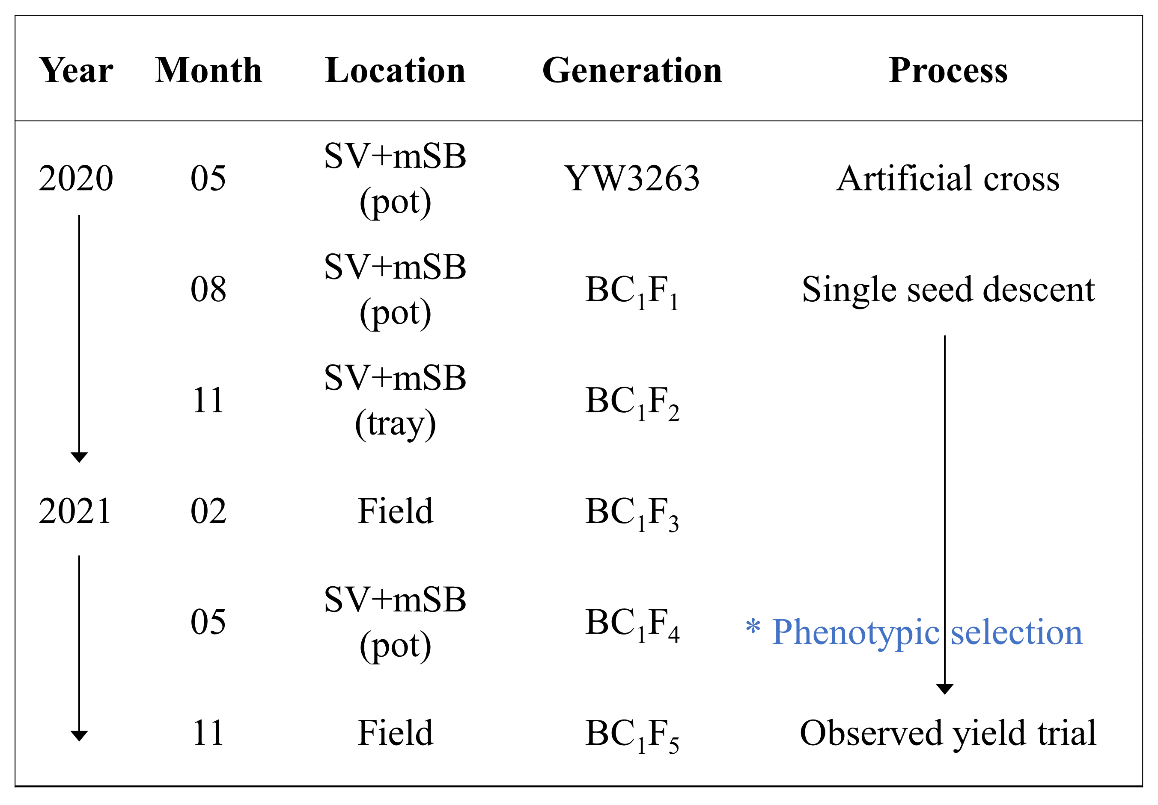


**Figure S3.** Timeline of the development of Joongmo2008*2/Tapdong BC_1_F_5_ lines and phenotypic selection under the speed vernalization + modified speed breeding (SV+mSB) system.
